# Study protocol of a co-created primary organizational-level intervention with the aim to improve organizational and social working conditions and decrease stress within the construction industry – a controlled trial

**DOI:** 10.1186/s12889-020-08542-7

**Published:** 2020-03-30

**Authors:** Emma Cedstrand, Anna Nyberg, Theo Bodin, Hanna Augustsson, Gun Johansson

**Affiliations:** 1 Unit of Occupational Medicine, Institute for Environmental Medicine, Karolinska Institutet, Stockholm, Sweden; 2 Stress Research Institute, Department of Psychology, Stockholm University, Stockholm, Sweden; 3 Center of Occupational and Environmental Medicine, Stockholm County Council, Stockholm, Sweden; 4 Department of Learning, Informatics, Management and Ethics, Medical Management Centre, Karolinska Institutet, Stockholm, Sweden

**Keywords:** Occupational health, Organizational level intervention, Process evaluation, Effectiveness evaluation, Co-creation, Construction industry

## Abstract

**Background:**

Within construction industry, physical work exposures have long been recognized as possible determinants for musculoskeletal disorders, but less attention has been given the increasing organizational and social work hazards and stress within this industry. There is to date a lack of knowledge about how to improve organizational and social working conditions and decrease stress within the construction industry.

**Methods:**

This paper outlines the design of a controlled trial to evaluate the effectiveness of a co-created organizational-level intervention with the aim to improve role clarity, quantitative demands, staffing, planning, team effectiveness, psychosocial safety climate and stress. Two regions (> 700 employees) within one large construction company in Sweden will participate as intervention and control group. Further we present the design of the process evaluation assessing fidelity, support from managers, readiness for change and contextual factors. We will utilize questionnaires, semi-structured interviews, observations and documentation as means for data collection, hence a mixed methods approach is applied.

**Discussion:**

The study is expected to contribute to the understanding of how adverse organizational and social working conditions and stress can be improved within the construction industry. By applying co-creation we wish to develop an intervention and implementation strategies that fit to the context, are in line with the needs of end-users and are supported by all management levels - all of which are highlighted features in successful workplace interventions.

**Trial registration:**

ISRCTN, ISRCTN16548039. Registered 12/02/20. Retrospectively registered

## Background

Working conditions are an important health determinant [1]. Many studies have identified the physical hazards of the construction trade and the following health effects [2]. However, there has been an intensification of work across the labor market over the past decades and psychosocial risk factors are common [3]. Also, within the construction industry has work become more stressful [4]. Hence, not only physical hazards but also the psychosocial work environment should be considered among construction workers [5]. There is, however, a dearth of knowledge on the relationship between adverse factors in the psychosocial work environment and mental health problems within male dominated industries in general [6] and in the construction industry specifically [7]. Further, the size and scope of the problem seem uncertain. Reports show that the industry has the second highest incidence rates regarding occupational mental health disorders in the Netherlands [7], while, on the other hand, in the UK, construction workers have a lower incidence of mental illness compared to workers in other industries [8]. Swedish statistics show similar figures as in the UK, however poor mental health as a cause of sick leave is increasing across the Swedish labor market, including the construction industry [9, 10]. Stress related diagnoses, such as acute stress reaction and burnout, are increasing the most [9].

A possible consequence of stress among construction workers is an increased risk of being involved in workplace accidents [11–13]. A Swedish report [11] shows that construction workers who report daily stress suffers a four times as high risk of being in a serious workplace accident compared to those reporting perceived stress seldom or never. Hence, understanding the relationship between psychosocial work conditions and stress and how these working conditions can be changed within the construction industry can lead to a decrease in both stress and workplace accidents. Further, several countries’ occupational health and safety legislation obliges employers to act against psychosocial risk factors that cause work stress [14]. In Sweden, the legislation on organizational and social work environment was sharpened in 2016 when new provisions were implemented, placing requirements on the employer regarding knowledge requirements, goals, workload, working hours and victimization.

One challenge is to identify the important psychosocial risk factors. A recent systematic meta-review [1] concludes that high job demands, low job control, role stress, bullying and low social support in the workplace are risk factors for common mental health problems. However, the root cause of psychosocial risks and work-related stress has been proposed to be the psychosocial safety climate [15] in the organization. Hence, this is also an important factor to examine.

Interventions to address work-related stress are increasing but are mainly secondary or tertiary [16, 17]. Secondary interventions are directed at individuals at risk of developing stress responses. Tertiary interventions focus on treating existing diagnosed conditions. Primary interventions on the other hand are preventive and aim to deal with organizational factors as causal stress agents. However, there is a dearth of knowledge on how to improve organizational and social working conditions and prevent poor mental health at the workplace [18]. Thus, to address this knowledge gap the focus of this project is on a primary organizational-level intervention. The present project is expected to contribute to the understanding of how adverse organizational and social working conditions can be improved.

It is argued that both effect and process evaluations are needed when evaluating complex organizational-level interventions [19–21] in order to gain a better understanding of the mechanisms of change. In the present study we will follow these recommendations. There are several theoretical frameworks deriving from different disciplines, for how to evaluate the implementation process [21–25]. In this study, the Medical Research Council (MRC) guidance on process evaluations [21] will mainly be used together with parts of the Framework for Evaluating Organizational-level Interventions by Nielsen and Randall [22]. The latter will be used as it has been developed specifically to evaluate organizational-level occupational health interventions and because using the same terminology as other researchers within the field has been recommended [26].

### The implementation process and co-creation

Conditions found to be crucial for successfully implementing change are: fit of the intervention in to the workplace context [22], integration of the intervention activities into already existing structures [27], and that the intervention build on a participatory approach for both the management and the target group for change, the so called end-users [28, 29]. Hence, it is important that the intervention is tailored for the specific group and context [30, 31]. Finally, the support from line and senior managers has been acknowledge as crucial for the implementation to succeed [22, 32, 33]. To meet these implementation conditions Leask et al. [34] recommend that researchers and different stakeholders from the organization undertaking the change should co-create the intervention. This process should for example include shared decisions on who should be included in the co-creation, problem formulation and goal setting. We will use co-creation in order to ensure relevance regarding content of the intervention and to enhance the implementation process variables described above.

### Aim and objectives

The aim of this project is to contribute to the knowledge on how to change adverse working conditions within the construction industry by evaluating a co-created intervention with the aim to improve organizational and social working conditions, enhance team-effectiveness and decrease stress.

The primary objective is to compare the effectiveness of a co-created intervention versus standard procedures on role clarity, quantitative demands, staffing, planning, team effectiveness, psychosocial safety climate and stress (effectiveness evaluation).

The secondary objective is to evaluate the implementation process regarding fidelity (adherence to the intervention) and barriers and facilitators to adherence. This will be done using mixed methods, that is, both qualitative and quantitative methods will be used to collect data.

## Methods

### Trial design

The study is a controlled trial with before and after measurements involving two regions (one intervention and one control region) and approximately 45 construction teams (projects). Randomization was not possible due to the fact that the intervention region wanted all groups (building projects) to receive the intervention. Instead we matched a control group (region). The criteria applied for matching were type of work (same branch) and region size (*N* > 300). Employees of the participating regions will be invited to complete outcome questionnaires during working hours at baseline and at 12 months follow-up. Our intention is to also include an 18-month follow-up questionnaire, however this has not yet been approved by the control region.

### Trial registration

The trial has been registered in the ISRCTN registry (16548039). Further, the study has been approved by the Swedish Ethical Review Authority (Reg. No. 2019–02662).

### Study setting and study population

The Swedish construction industry occupy around 300,000 individuals in Sweden. The gender distribution is uneven and most employees are men [11]. Approximately 15% are employed in large companies and the studied organization is one of the largest construction companies in Sweden. The company operates internationally but the focus of this project is on employees and work sites located in Sweden.

#### Recruitment of regions

Two large construction companies in Sweden were contacted and after a few meetings one of them agreed to participate in the study. A short listing of eligible branches and regions was conducted in collaboration with representatives from the company. The building construction branch was chosen as the context for the intervention. The national manager of health and safety took on the responsibility to inform the regions about the study and look for potential participants. One region (employees = 360) applied to take part in the study with one condition, the whole region should be included in the intervention. Hence, this became the intervention group. The matched control region (employees = 450) accepted to participate after discussions in their highest management team.

#### Participant eligibility criteria

Participants employed by the included regions were eligible to participate in the study. This includes both blue- and white-collar workers within the building projects but also the white-collar workers belonging to the supporting group (operational support). Employees reporting to managers outside the region, the regional manager, the districts managers, and the regional managers staff were excluded from the effectiveness evaluation since the interventions do not target their working situation. However, they are important stakeholders in relation to the implementation process and therefore included in the process evaluation.

### Intervention development and planning

The project includes three phases and follow the evidence based psychosocial risk management approach [35], which can be summarized in five steps: preparation, screening, action planning, implementation and evaluation. During preparation we formed an operative steering group consisting of the Human Resource (HR) generalist, the Health and Safety manager, the manager of development and the project leader from the research team. The regional manager was assigned as project owner together with the highest management team. In order to reach a buy in among the senior management all suggestions were presented to the highest management team who agreed to include the prioritized outcomes and intervention activities in the business case for 2020. An already existing work group, called the Health and Safety team, was chosen as the co-creation group. The group consists of representatives from different levels (e.g. first-line managers, site managers) and districts within the region, union representatives together with the HR generalist, the health and safety manager, the manager of operational support and the manager of development. Screening included a formative evaluation to assess the current working conditions. We carried out interviews (*n* = 25) and a survey in order to answer the questions: *What works well?* and *What needs to be improved*? regarding the organizational and social work environment. In order to give the intervention and the control group similar conditions, the survey was also conducted in the control group, however no feedback of the results was given to the control group.

In the second phase (action planning) researchers and the Health and Safety team co-created the outcomes, the intervention components and the implementation strategies. Hence, a co-created program logic was created, which is a recommended model [36]. We also discussed and formulated suggestions for how to design the implementation process. During the third phase the intervention will be implemented and evaluated.

#### Program logic and interventions

**Fig. 1:**
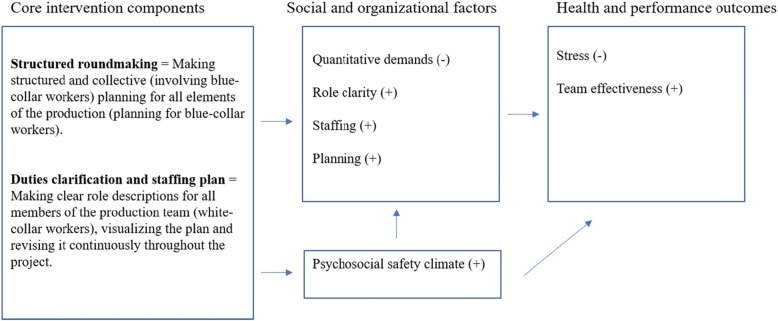
Program logic for the intervention. (+) = an increase in the outcome, (−) = a decrease in the outcome

In the co-creation group, we produced a co-created program logic working our ways backwards in the logic model, see Fig. 1. The long-term outcomes, stress and team effectiveness, were firstly chosen, then the short-term outcomes and finally the intervention components identified in relation to the outcomes. The short-term outcomes were chosen after thorough investigation of the results from the needs assessment (interviews and survey). This was an iterative process going on for 4 months. Hence, the intervention components fit well into the context, senior management supports them, and the co-creation group (Health and Safety team) has been involved in the discussion and decision-making regarding outcomes and interventions.

#### Implementation strategies

This is an intervention study, however since research has shown the great importance of a well-planned and executed implementation process [26] we decided, not only to make a program logic for the intervention but also to include a clear description of what implementation strategies that will be used. It has been suggested that the choice of implementation strategies should be theory-based, and target identified barriers [26]. Therefore, we utilize the Com-B model [37] to address barriers and facilitators and to choose implementation strategies to target these barriers. However, some of the implementation strategies have been chosen by the research team given barriers and facilitators identified in earlier research, such as lack of fit to the context, and lack of support from managers [38]. See Table 1. To facilitate comparison between studies, we used the terminology of Powell et al. [40] to describe the implementation strategies.

**Table 1:** Description of implementation activities, content according to Powell (implementation strategies), barriers to target and theory used

Activity	Implementation strategies included	Barriers to target	Who identified the barrier?	Theory
Co-creation	Use advisory boards and workgroups	Lack of fit into the context	Literature: [33, 38] Lack of support from managers	
		Lack of support from managers		
	Conduct local consensus discussions			
		Lack of integration in existing structures	[22, 31] Lack of fit into the context	
			[27] Lack of integration in existing structures	
Formative evaluation	Conduct local needs assessment	Lack of fit into the context	Literature: [22, 39] Lack of readiness for change	
		Lack of readiness for change among end-users		
Feedback of results and rational to interventions	Conduct educational meetings	Lack of understanding	The co-creation team	Lack of motivation COM-B model
Develop educational materials	Develop educational materials	Lack of competence	The co-creation team	Capability and opportunity COM-B model
Learning collaborative	Identify early adopters	Lack of motivation	The co-creation team	Lack of motivation COM-B model
	Shadow other experts	Lack of competence		
	Visit other sites	Lack of understanding		
	Create a learning collaborative			

#### Sample size /power calculations

Since the size of the intervention and control region was given, we performed power calculations. However, since the outcomes were chosen through co-creation later in the process we elaborated with different mean differences (5, 7, 9 and11) and different standard deviations (SD) 10, 15, 20 and 30. Based on a level of significance (α) of 0.05, results show a desired statistical power (1-β) of 0.9 given a mean difference of 5 and a SD not higher than 15. A SD of 20 with the same mean difference gives a statistical power (1-β) of 0.8. Given a higher mean difference between the intervention and control group, power increases, even with larger standard deviations. A mean difference of 9 and a SD of 30 gives a statistical power (1-β) of 0.8.

### Data collection (effectiveness evaluation)

All primary and secondary outcomes will be assessed at baseline and at a 12-month follow-up. An online survey will be distributed during working hours.

#### Primary outcome

The primary outcome is *stress* assessed with three items (e.g. How often have you had problems relaxing?) preceded by “These questions are about how you have been during the last 4 weeks” from the Copenhagen Psychosocial Questionnaire (COPSOQ), version III [41]. COPSOQ covers a broad range of organizational and social work conditions. The instrument is well-established and was developed for use in occupational risk assessment and research on work and health. The response categories for the three stress items range from (1) “all the time” to (5) “not at all”.

#### Secondary outcomes

*Quantitative demands* assessed with three items (e.g. Do you get behind with your work?) from COPSOQ III with response categories ranging from (1) “always” to (5) “never/hardly ever”.

*Role clarity* assessed with three items (e.g. Does your work have clear objectives?) from COPSOQ III with response categories ranging from (1) “to a very large extent” to (5) “to a very small extent”.

*Psychosocial safety climate (PSC)* is assessed with four validated [42] items (e.g. Senior management considers employee psychological health to be as important as productivity). The response categories range from (1) strongly disagree to (5) strongly agree.

*Team effectiveness* is assessed with four items (e.g. How effective is your team in making use of the skills of the different team members?) from a scale developed by Maynard [43]. The response categories ranging from (1) “not good at all” to (5) “very good”. This scale has not been validated in Swedish.

*Planning* is assessed with one item (Do you experience the work at your workplace as well-planned?), previously used in a Swedish report [11] looking at the relation between serious workplace accidents and the work environment.

*Staffing* is assessed with two self-constructed items (e.g. Is the staffing at your workplace sufficient in terms of number of individuals?). The response categories ranging from (1) “to a very high extent” to (5) “to a very low extent”.

### Data collection (process evaluation)

*Fidelity* will be operationalized as adherence to the intervention. To assess this, we will ask all managers who are supposed to make the behavior change to rate to what extent they are undertaking the intervention components (specified behaviors) prior to the implementation and again after the project has finished. Hence, the intervention components are not completely new, rather they have a potential of improvement. To assess adherence to duties clarification and staffing we will use a questionnaire comprising 7 items (e.g. We have performed a duties clarification regarding the work tasks in the project). The questionnaire to assess structured roundmaking comprise 8 items (e.g. Structured roundmaking is a part of the weekly schedule for the first line manager). The response categories for both scales ranging from (1) “to a very high extent” to (5) “to a very low extent”. All items build on the company’s own standards for evaluation. In addition to this, one of the intervention components (structured roundmaking) will be assessed using observations. We will do this to minimize the risk of social desirability [44] as observation is a more objective and valid way of evaluating behavioral changes.

*Readiness for change* and *Support from managers* will be assessed using the validated Intervention Process Measure (IPM) [45]. Readiness for change will be assessed before the implementation. The measure consists of five subscales of which we will use two: line manager attitudes and actions (Support from managers*)* and employee readiness. Randall (2009) recommends tailoring of the items to the specific contexts, which we did by specifying the interventions (structured roundmaking and duties clarification). Hence, readiness for change is assessed with four items (e.g. “I am ready to accept the changes brought about by the implementation of structured roundmaking”). Support from managers is assessed with seven items (e.g. “My immediate manager was positive about the implementation of structured roundmaking”). The response categories range from (1) “strongly agree” to (5) “strongly disagree”, Likert-type scales.

#### Barriers and facilitators to adherence

This will be explored using semi-structured interviews with different stakeholders (e.g. managers supposed to employ the intervention components, senior managers, safety representative) during the intervention and at the end or after completion. Informants will be chosen from the intervention projects with the aim to get comprehensive and rich data from different levels within the company. Hence the sampling strategy will be stratified purposeful using the pre-defined criterias nested and multi-level [46]. Three to four informants within each district (a total of 4 districts) will be asked to participate in the qualitative study. A minimum of 12 informants will be included following the recommendations of Onwuegbuzie (2007).

*Dose* (the quantity of implementation strategies fulfilled) *and reach* (workers’ participation in the completed activities) will be documented using attendant lists and a logbook.

#### Risk of contamination

To assess whether and to what extent the control region works with improvements in the chosen intervention components we will conduct semi-structured interviews with relevant stakeholders from the control region. We will also study their business case for 2020 to investigate the control region’s objectives and activities regarding health and safety.

### Statistical methods (intervention effectiveness)

To evaluate the effect of the intervention we will examine between group differences over time. The analyses will consider the clustering of observations of workers within the working team (project), as well as the repeated measurements within each worker. Intention-to-treat analyses will be used and where relevant, compared to per-protocol analyses. Adjusted models will be applied if potential confounders are unevenly distributed and if this is likely to affect the results when the two groups (intervention and control) are compared.

### Qualitative analysis (interviews and observations)

Regarding the qualitative data we will perform thematic analysis [47]. The interviews and the observations will be digitally recorded an transcribed verbatim. Field notes from the observations will also be included in the analysis. The first step of the analysis will be to listen to the recordings and read through notes and transcripts to familiarize with the data. A data-driven coding process will follow, which is performed by two investigators independently. The coding will set the ground for the initial theme creation, which will then be discussed in the research group to enhance credibility. To further understand the data and highlight findings the researchers, if found appropriate, will revisit the literature (theory) and employ an iterative process between the data and the literature (theory) according to the tin-opener approach [48].

## Discussion

This paper outlines the design of a controlled trial testing the effectiveness of a co-created behavior change intervention with the aim to improve organizational and social working conditions, team effectiveness and decrease stress. To the best of our knowledge not many primary organizational-level intervention studies with the aim to improve organizational and social working conditions and decrease stress have been conducted within the construction industry. With this study we wish to add to the scientific literature with knowledge about how adverse psychosocial working conditions can be improved within the construction industry.

Given the importance of making the intervention fit to the context, discussed in earlier research, our intention was to, instead of viewing interventions as discrete packages of components isolated from their contexts [31], include the system into which we wanted to introduce change in our intervention. Another factor described in the literature to be important for the success of interventions is to let the receivers of the intervention participate in designing it and deciding on its content. Most interventions within the literature are described as participatory, however a clarification of in what way the intervention is participatory is often lacking [29]. Given the importance of a contextual fit and a participatory approach we chose to focus on co-creation as a means to reach these goals. In practice this meant that representatives from the studied organization (end-users and providers) were invited to discuss and decide, not only the content (intervention components) but also the process (implementation strategies). In line with recommendations in the literature [23, 40] we carefully selected and defined the implementation strategies suitable for the identified barriers and facilitators [23, 40]. In sum, we believe that by designing an intervention study with co-creation of the logic model and implementation strategies, we have taken thorough action in order to avoid implementation failure, which has been proposed to be of high priority for success with intervention studies [23].

One limitation is the possible risk of selection bias because randomization was not possible. It is likely that the included intervention group represents a motivated group with a high interest in improving the psychosocial work environment as they volunteered for the study. Co-creation also has its benefits and constraints. The research group somewhat loses control, in this study over the outcomes and intervention components selected, as it is in the nature of co-creation to let the end-user prioritize this. In this study the choice of intervention components fell on improving core tasks, rather than testing new routines. This implies a risk of contamination between the intervention and the control group. However, this will be monitored using interviews with relevant stakeholders within the control region, keeping track of whether, and if relevant how, they are focusing on improving the same routines.

The study also has several strengths. First, the co-creation of the outcomes, intervention components and the implementation strategies have enhanced the fit into the context and ensured a buy-in from senior management. The fact that the outcomes and the intervention components have been included in the business case for 2020 enhances the possibility that the project will be prioritized. It is, to our knowledge, the first time this is done in an occupational health organizational-level intervention study. Furthermore, the structured approach to identifying barriers and facilitators and thereafter choosing theory-driven (COM-B) implementation strategies is a recommended procedure [26]. Last, the evaluation of both effectiveness and the implementation process is a recommended and preferable strategy [21] in order to be able to understand why (or why not) and in what way the intervention was effective.

## Data Availability

The datasets generated and analyzed during the current study are not publicly available due to legal restrictions.

## Abbreviations

MRC: Medical research council
HR: Human resources
PSC: Psychosocial safety climate
COPSOQ: Copenhagen psychosocial questionnaire
